# An Economical and Flexible Dual Barcoding, Two-Step PCR Approach for Highly Multiplexed Amplicon Sequencing

**DOI:** 10.3389/fmicb.2021.669776

**Published:** 2021-05-20

**Authors:** Petra Pjevac, Bela Hausmann, Jasmin Schwarz, Gudrun Kohl, Craig W. Herbold, Alexander Loy, David Berry

**Affiliations:** ^1^Joint Microbiome Facility of the Medical University of Vienna and the University of Vienna, Vienna, Austria; ^2^Division of Microbial Ecology, Department of Microbiology and Ecosystem Science, Centre for Microbiology and Environmental Systems Science, University of Vienna, Vienna, Austria; ^3^Department of Laboratory Medicine, Medical University of Vienna, Vienna, Austria

**Keywords:** microbiome, high-throughput amplicon sequencing, standardized workflows, 16S rRNA gene, unique dual barcoding

## Abstract

In microbiome research, phylogenetic and functional marker gene amplicon sequencing is the most commonly-used community profiling approach. Consequently, a plethora of protocols for the preparation and multiplexing of samples for amplicon sequencing have been developed. Here, we present two economical high-throughput gene amplification and sequencing workflows that are implemented as standard operating procedures at the Joint Microbiome Facility of the Medical University of Vienna and the University of Vienna. These workflows are based on a previously-published two-step PCR approach, but have been updated to either increase the accuracy of results, or alternatively to achieve orders of magnitude higher numbers of samples to be multiplexed in a single sequencing run. The high-accuracy workflow relies on unique dual sample barcoding. It allows the same level of sample multiplexing as the previously-published two-step PCR approach, but effectively eliminates residual read missasignments between samples (crosstalk) which are inherent to single barcoding approaches. The high-multiplexing workflow is based on combinatorial dual sample barcoding, which theoretically allows for multiplexing up to 299,756 amplicon libraries of the same target gene in a single massively-parallelized amplicon sequencing run. Both workflows presented here are highly economical, easy to implement, and can, without significant modifications or cost, be applied to any target gene of interest.

## Introduction

Sequencing of phylogenetic and functional marker gene amplicons is a widely-used and cost-effective approach for characterizing the composition and dynamics of microbial communities from virtually any type of clinical or environmental sample (Hamady and Knight, 2009; Gilbert et al., 2010). The rapid advances in both quality and yield of short-read sequencing technologies has made amplicon sequencing accessible and affordable to the broader scientific community, and has led to the ubiquitous implementation of amplicon-based microbial community profiling approaches in microbiome research. Simultaneously, the increased accessibility and the ever-growing application scope of amplicon sequencing has led to a surge in attempts to develop standardized procedures and best practices for the generation and analysis of sequencing data in microbiome studies (reviewed in Knight et al., 2018). However, standardization of such workflows is not trivial. The main reason for this is the number of individual steps in an amplicon sequencing workflow: sample collection, preservation and storage, nucleic acids extraction, target gene selection and amplification, sequencing, and finally *in silico* data processing, analysis and interpretation. For each step of the workflow several options are available, resulting in a multitude of possible workflow variations and combinations. How different approaches for many of these steps (e.g., nucleic acids extraction method or primer selection for target gene amplification) affect amplicon sequencing results has been critically reviewed (e.g., Sinha et al., 2017; Pollock et al., 2018; Allaband et al., 2019). Notably, a concern in many microbiome studies is so-called batch effects, a term commonly used to refer to artifacts and confounding factors originating from biological, technical, and computational sources of variation in handling and processing of sample or sample groups that are ultimately compared to each other (Wang and LêCao, 2020). It is thus essential for any workflow to minimize possibilities for the introduction of such batch effects.

While a universal “best practice guideline” to amplicon sequencing based microbiome analysis is not feasible or even desirable—in part due to varying sample- and study-specific requirements, but also due to conflicting experimental data (reviewed in Pollock et al., 2018)—one consensus does exist: methodological consistency and minimized variance in sample processing within a single study is of utmost importance to the validity of any dataset, and is essential in order to avoid or minimize erroneous interpretations of microbiome data.

As amplicon sequencing has become the most widely-used method for microbial community profiling, economical workflows that allow for very high throughput are in demand. Simultaneously, the decreasing costs of sequencing and rapid advances in sequence quality achieved in recent years has put into focus the need for improvements in amplicon sequencing workflows that ensure maximal data quality. Considering sequence data processing, the improved quality of data has led to the transition to sequence data analysis on the level of biologically more-meaningful amplicon sequence variants (ASVs) (Callahan et al., 2017), which are gradually replacing the widely-used but largely arbitrarily-defined operational taxonomic unit (OTU) approach. On the side of data generation, the issue of index or barcode crosstalk, which can lead to misassignment of sequences in massively parallel sequencing approaches, has received considerable attention (summarized in MacConaill et al., 2018). As millions of sequence reads can be generated in a single sequencing run, large numbers of samples are typically pooled prior to sequencing to assure cost-efficient and scalable workflows. Prior to pooling, individual samples are tagged with sample specific DNA fragments, referred to as sample indexes or barcodes. These indices, or barcodes, are used to computationally separate (demultiplex) pooled samples after sequencing. However, a risk of sequence read misassignment during de-multiplexing is inherent to these approaches. The exact source of crosstalk can in most cases not be precisely identified, as crosstalk is usually caused by a combination of barcode or index oligonucleotide contamination at synthesis or during handling (Quail et al., 2014), sequencing errors, and to a lesser extent barcode or index hopping (i.e., switching of barcodes or indexes between different sequencing libraries due to unknown causes). The risk of crosstalk can be further decreased by applying sample barcoding strategies which incorporate multiple differences that discriminate between samples. As a consequence, dual indexing approaches—where each sample is tagged with two unique indexes or barcodes—are now considered the gold standard for applications in which tens to hundreds of sequencing libraries are multiplexed in a single sequencing run (Esling et al., 2015). While challenging to quantify, the magnitude of sample crosstalk in single barcoding approaches (based on erroneous pairing for barcodes and indexes in dual indexing approaches) has been reported to reach up to 0.3% (Kircher et al., 2012). This frequency of sequence misassignment in amplicon sequencing workflows employing single barcoding is not a major concern if the primary aim of the amplicon analysis is community profiling. However, such missasignments do become a considerable risk if low abundance amplicon sequence variants (i.e., the rare biosphere) are of scientific interest to a study.

In most amplicon sequencing workflows, sample-specific barcodes are introduced at the step of sequencing library preparation, meaning that each amplicon sample is an individual sequencing library in a multiplexed run. However, as the number of DNA sequences that can be used as indices is limited (Faircloth and Glenn, 2012), so is the number of samples that can be multiplexed by this approach. Furthermore, sequencing library preparation is one of the more costly and time-consuming steps of an amplicon sequencing workflow, and thus is a limiting step for economical sample indexing or barcoding approaches. A more economical and flexible approach of sample barcoding is a second PCR step, performed after the target gene amplification PCR and before library preparation, that has been developed and successfully applied to a variety of samples (Herbold et al., 2015). Here, we further optimized this two-step PCR approach to enable either highly-accurate assignment of sequences to source samples *via* unique dual barcoding (UDB), or to allow for magnitudes higher levels of sample multiplexing *via* combinatorial dual barcoding (CDB).

## Materials and Methods

### Mock and Environmental Reproducibility Controls

A commercially-available mock community (ZymoMock, ZymoBIOMICS Microbial Community DNA Standard II, D6311; Supplementary Table 1) and a complex environmental sample (Soil) was used to evaluate the different sample barcoding setups with respect to extent of barcode crosstalk, and the effects of variability in amplicon preparation, library preparation, and sequencing on the quality and reproducibility of amplicon sequencing results (i.e., batch effects). The mock community was ordered as a DNA standard, while environmental DNA was extracted from a peatland soil using a phenol-chloroform based method after mechanical cell lysis (bead-beating) (Hausmann et al., 2016).

### Target Gene (First-Step) PCR

**FIGURE 1:**
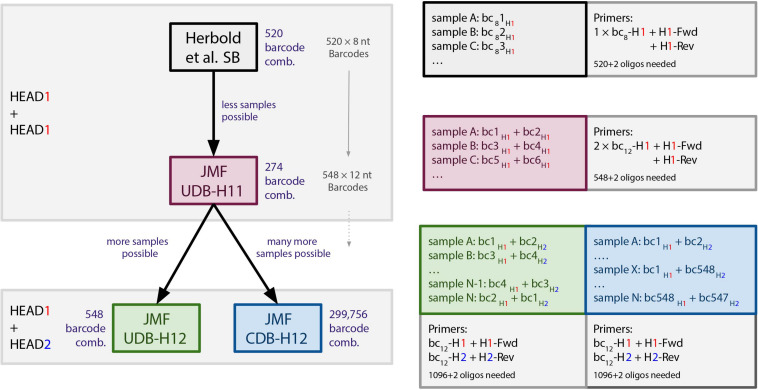
Overview of barcoding systems evaluated in this study. bc_8_ = 8 nt long barcodes (520 available). bc/bc_12_ = 12 nt long barcodes (548 available). Fwd/Rev, forward/reverse primer. SB, single barcoding. UDB, unique dual barcoding. CDB, combinatorial dual barcoding.

In the first amplification step, the V4 or V3–V4 regions of the 16S rRNA genes were amplified with the 515F/806R or 341F/785R primers (Table 1), modified to contain either the same 16 nt head sequence (**H1**: *5*′-GCT ATG CGC GAG CTG C-*3*′; Herbold et al., 2015) at the 5′ end of both the forward and reverse primer, or the H1 sequence at the 5*′* end of forward primer, and a newly designed 16 nt head sequence (**H2**: *5*′-TAG CGC ACA CCT GGT A-*3*′) at the 5*′* end of the reverse primer (Figure 1). PCR reactions for each sample were set up as triplicate 25 μl reactions using the DreamTaq Green PCR Master Mix (ThermoFisher), with 0.25 μmol L^–1^ of forward and reverse primer each and 2 μl DNA template. Four PCR negative controls (PCR with nuclease free water as template) were routinely performed per 90 samples. Thermal cycling was performed as shown in Table 1. PCR products were subsequently analyzed by gel electrophoresis either on in-house cast 2% low electroendosmosis (LE)-agarose gels or using an E-Gel system (ThermoFisher) with precast 2% E-Gel 96 Gels with SYBR Safe DNA Gel Stain (ThermoFisher). Thereafter, triplicate first-step PCR reactions were pooled and purified and normalized with the SequalPrep Normalization Plate Kit (Invitrogen) following the manufacturers’ instructions. This clean-up and normalization approach removes DNA fragments shorter than 100 bp (i.e., primer dimers) and adjusts the amount of recovered DNA per reaction to a maximum of 25 ng prior to use as a template for barcoding (second-step) PCRs. The PCR product is combined with a kit-specific binding buffer and transferred into a SequelPrep normalization plate, the walls of which are coated with a DNA binding solid phase capable of retaining a maximum of 25 ng DNA. Unbound PCR products (including short fragments, which cannot bind to the solid phase) and buffer are removed, and the now normalized and cleaned PCR product is detached from the DNA-binding solid phase with a kit-specific elution buffer.

**TABLE 1:** 16S rRNA gene-targeted oligonucleotide primers used in this study.

Primer name	Primer sequence (5′–3′)	Cycling conditions	References
**V4 region of 16S rRNA gene**			
515F Parada	GTG YCA GCM GCC GCG GTA A	94°C for 3 min 30 cycles of: • 94°C for 45 s • 52°C for 60 s • 72°C for 90 s 72°C for 10 min	Parada et al., 2016
806R Apprill	GGA CTA CNV GGG TWT CTA AT		Apprill et al., 2015

**V3–4 region of 16S rRNA gene**			

341F	CCT ACG GGN GGC WGC AG	94°C for 3 min 30 cycles of: • 95°C for 30 s • 55°C for 30 s • 72°C for 60 s 72°C for 10 min	Herlemann et al., 2011
785R	GAC TAC HVG GGT ATC TAA TCC		Herlemann et al., 2011

### Barcoding (Second-Step) PCR

In the second amplification step, either a single barcoding primer, consisting of a 8 nt barcode (bc8) and H1 (*5*′-BC8_1-H1-*3*′; see also Herbold et al., 2015); a single barcoding primer, consisting of an 12 nt barcode (bc12) and H1 (*5*′-BC12_1-H1*-3′*); or two barcoding primers each consisting of a distinct 12 nt barcode and one of the two used 16 nt head sequences (*5*′-BC12_1-H1-*3*′ and *5*′-BC12_2-H2-*3*′) were used for amplification (Figure 1). The 8 nt barcodes (*n* = 520; Supplementary Table 2) used were the same ones as used by Herbold and colleagues (Herbold et al., 2015, previously published in Hamady et al., 2008). The 12 nt barcodes (*n* = 549, Supplementary Table 3) were selected from a list of 959 12 nt barcodes used in the Earth Microbiome Project (Walters et al., 2016), based on following criteria: Hamming distance ≥ 4 from one another [as determined by the R-package DNABarcodes (Buschmann and Bystrykh, 2013)], and together having a uniform base distribution at each barcode base position. All oligonucleotide barcodes used for this study were synthesized and HPLC purified by and purchased from Microsynth (Balgach, Switzerland). Due to the nature of the barcoding PCR setup, 548 of these 549 12 nt barcodes are currently in use at the JMF (Supplementary Table 3).

Barcoding PCR reactions were set up as a single 50 μl reaction using the DreamTaq PCR Master Mix (ThermoFisher), with 0.8 μmol L^–1^ of each used barcoding primer and 10 μL of purified and normalized first-step PCR product as template. The barcoding PCR was performed with following thermal cycle conditions: initial denaturation at 94°C for 4 min; 7 cycles of denaturation at 94°C for 30 s, annealing at 52°C for 30 s, and elongation 72°C for 60 s; followed by a final elongation step at 72°C for 7 min. Thereafter, samples were again purified and normalized with the SequalPrep Normalization Plate Kit as described above.

### Sequencing Library Preparation and Sequencing

Barcoded, purified, and normalized PCR amplicons were multiplexed and concentrated with the innuPREP PCRpure Kit (Analytik Jena). In the here analyzed datasets, up to 546 distinct amplicon libraries, i.e., samples amplified with the same first-step primers, were multiplexed in a single amplicon pool (Table 2). Thereafter, the amplicon pool fragment size was verified on a D1000 ScreenTape Assay using a TapeStation 4200 (Agilent), and the DNA concentration in the amplicon pool was quantified with a Quant-iT dsDNA Assay (ThermoFisher) on a Qubit 4 Fluorometer (ThermoFisher). Sequencing libraries were prepared and indexed by adapter ligation and PCR (8 cycles) using the TruSeq Nano DNA Library Prep Kit (Illumina) and TruSeq DNA Single Indexes Set A or B (Illumina), excluding the DNA fragmentation and clean-up of fragmented DNA steps, and otherwise following the manufacturer’s instructions. Sequencing library size and the efficiency of unligated adaptor removal were verified on a D1000 ScreenTape Assay using a TapeStation 4200, and the sequencing library concentration was determined with a Quant-iT dsDNA Assay on a Qubit 4 Fluorometer. For each sequencing run, 2–5 amplicon pool libraries (in total up to 1,092 distinct amplicon libraries) were combined and sequenced in 2 × 300 cycle paired-end mode on an Illumina MiSeq using the MiSeq Reagent kit v3 (Illumina) with 6 nt library indexes (DNA Single Indexes Set A or B, Illumina). To achieve sufficient variability during the first five sequencing cycles, which is necessary for efficient sequence cluster identification and phasing/prephasing calibration during Illumina sequencing (Fadrosh et al., 2014), we spiked in 1–7 random shotgun genomic or metagenomic sequencing libraries (to an abundance of 9–21%) and 1% PhiX control to each sequencing run.

**TABLE 2:** Overview of all analyzed runs separated by amplicon pools. Yield is given in read pairs and corresponds to Figure 2.

Run	Pool	Barcoding	Primers	Libraries	Total read pairs	Barcode-sorted read pairs	Final read pairs	NC	Mock	Soil	Note
C64CL	1	SB	V3–4	184	1 922 856	973 290	865 536				
CGPPL	2	CDB-H12	V4	478	17 073 818	10 267 424	7 641 530	20		5	
CBPFC	3	UDB-H11	V3–4	264	5 583 678	2 825 700	2 118 627	10	3		
	4	UDB-H11	V3–4	264	5 352 498	2 683 767	2 263 560		3		
	5	SB	V3–4	64	1 841 019	1 062 035	970 704				
CGTHM	6	CDB-H12	V3–4 (and V4)	293	6 650 379	3 389 426	581 522	12	2		Contains nine V4 amplicons. All controls are from the V3–4 primer set.
	7	CDB-H12	V4	480	11 813 225	7 275 537	1 367 788	20	5	3	
CBM45	8	CDB-H12	V4	200	5 527 705	2 724 523	1 952 019	9	2	2	
	9	CDB-H12	V4	288	11 337 538	5 656 339	4 042 682	12	3	2	
	10	SB	V3–4	25	239 984	142 451	93 834				
	11	SB	V3–4	25	346 018	184 313	131 040				
CL24D	12	UDB-H11	V3–4	268	NA	NA	NA	10	3		56 non-standard amplicons were included in this pool, therefore yield data was calculated.
	13	UDB-H11	V3–4	264	3 228 166	1 563 877	1 382 073	11	3		
	14	UDB-H11	V3–4	144	4 275 834	2 142 075	1 357 170	7	2		
CBJCM	15	UDB-H11	V3–4	268	NA	NA	NA	10	3		56 non-standard amplicons were included in this pool, therefore yield data was calculated.
	16	UDB-H11	V3–4	264	1 811 989	810 445	630 204	11	3		
	17	UDB-H11	V3–4	144	1 834 298	854 991	468 849	7	2		
CL5WG	18	UDB-H12	V4	288	7 767 824	4 221 708	3 508 840	12	2	2	
	19	UDB-H11	V4	173	3 991 281	2 023 746	1 458 385	7			
	20	UDB-H12	V4	296	5 934 497	3 413 834	2 853 596	13	3	3	
CL87D	21	UDB-H12	V4	181	6 785 009	3 720 004	2 792 137	8	2	2	
CRRJM	22	UDB-H12	V3–4	268	5 531 591	3 345 618	2 499 843	11	3		
	23	UDB-H11	V4	235	3 933 425	1 901 242	1 531 626	9	3		
CRRCC	24	UDB-H12	V4	476	6 042 832	3 200 840	2 484 142	20	5		
	25	UDB-H12	V4	441	NA	NA	NA	20	5	3	20 non-standard amplicons were included in this pool, therefore yield data was calculated.
CWL3Y	26	UDB-H12	V3–4	86	10 009 360	5 472 947	4 885 729	4	1		
	27	SB	V3–4	36	447 184	272 896	241 612				
	28	SB	V3–4	16	381 340	217 329	173 503				
	29	SB	V3–4	14	279 219	155 500	124 336				
CRRK3	30	UDB-H12	V3–4	546	6 510 999	4 341 343	3 412 042	23	6		
	31	UDB-H12	V3–4	546	7 659 918	5 040 085	4 010 843	23	6		

*Number of total libraries, NC (negative controls), Mock (ZymoMock), and Soil (environmental control sample) libraries in each amplicon pool given.*

### Sequence Processing and Analysis

Individual amplicon pools were extracted from the raw sequencing data using the FASTQ workflow in BaseSpace (Illumina) with default parameters, allowing one mismatch for the 6 nt library indexes, followed by filtering for PhiX contamination with BBDuk (BBTools, Bushnell B^1^). Further demultiplexing of each amplicon pool library into single amplicon libraries was performed with the python package demultiplex (Laros JFJ^2^) allowing one mismatch for the 12 nt barcodes, and two mismatches for head sequence (H1 and H2) and primers (Table 1). Barcodes, linkers, and primers were trimmed off using BBDuk. SB datasets were analyzed as described in 14.

ASVs were inferred using the DADA2 R package version 1.14.1 Callahan et al. (2016a) with R version 3.6.1 (R Core Team, 2021) applying the recommended workflow (Callahan et al., 2016b). Therefore, 16S rRNA region V4/V3–4 amplicon FASTQ reads one and two were trimmed at 220/230 nt and 150/220 nt with allowed expected errors of 2/4 and 2/6, respectively. If not noted otherwise, ASV inference was performed in pooled mode using all amplicon libraries per sequencing run. Since error modeling in DADA2 can only be performed on sequencing data from the same run, results from separate sequencing runs were merged in R during post processing. All downstream analyses were performed in R version 4.0.2 (R Core Team, 2021).

The theoretical distribution of 16S rRNA genes for the ZymoMock, as specified by the provider (ZymoBIOMICS), was used to simulate count data. To this end, provided relative abundances of each 16S rRNA gene per species were used as probabilities in the R function rmultinom with a size of 2,438 simulated read pairs (100 replicates). All genomes of the bacterial mock community members contain multiple rRNA operons. In cases where the 16S rRNA gene copies within a genome were not identical over the amplified regions, they were treated as separate simulated ASVs (Supplementary Table 1). Inferred ASVs from amplicon libraries generated form the ZymoMock were matched to mock community members using the 16S rRNA gene sequences provided by the manufacturer (ZymoBIOMICS) as database for blastn (Nucleotide-Nucleotide BLAST version 2.10.1+) allowing for one mismatch.

Alpha diversity metrics, Bray–Curtis dissimilarity, and PCoAs were calculated using the R package ampvis2 version 2.6.5 (Andersen et al., 2018) with the R package vegan version 2.5–6 (Oksanen et al., 2020). All statistical comparisons between groups were performed using an ANOVA with post-hoc test TukeyHSD in R. Adjusted *P*-value significance codes used in all figures are ^∗^<0.05, ^∗∗^<0.01, ^∗∗∗^<0.001, and ^****^<0.0001.

#### Data Availability

Resulting datasets are deposited under the BioSample accession number SAMN16987472 (negative controls), SAMN13555796 (ZymoMock), and SAMN13555843 (environmental sample). Individual SRA accession numbers used in analyses are listed in Supplementary Table 4.

## Results and Discussion

### Combinatorial Dual and Unique Dual Barcoding Setups for Optimizing Read Assignment Accuracy and Amplicon Multiplexing Throughput

In this study, we extended upon a previously-published approach that utilizes single barcoding (SB) in a two-step PCR-based amplicon barcoding and sequencing workflow (Herbold et al., 2015). We evaluated two dual-barcoding strategies: unique dual barcoding (UDB), to minimize crosstalk between samples multiplexed in a single amplicon pool library, and combinatorial dual barcoding (CDB), to increase the number of amplicon libraries (i.e., samples) that can be multiplexed into a single amplicon pool library to hundreds of thousands.

The SB setup relies on the use of a single 16 nt head sequence (H1) modification of both the forward and reverse primers, as well as barcoding of the generated amplicon with one unique 8 nt barcode (bc8)-H1 fusion primer (Figure 1). With the SB approach, 520 samples can be targeted with the same primer pair, and the resulting 520 amplicon libraries can be multiplexed into one amplicon pool library (520 × *n × m* samples, where *n* is the number of distinct target-gene primer pairs used and *m* is the number of individual amplicon pool libraries combined on a sequencing run). As previously outlined (Kircher et al., 2012; Esling et al., 2015), crosstalk risks associated with all SB protocols also apply to this approach.

The UDB and CDB approaches we implemented rely on a different set of barcode sequences than the bc8 used in the SB setup. For dual barcoding, we used a subset of 548 unique 12 nt barcodes (bc12) that were selected from a list of 959 bc12 used in the Earth Microbiome Project (Walters et al., 2016), as further detailed in the methods. The advantage of bc12 over bc8 is the increased dissimilarity between individual barcodes, which increases the median Hamming distance from 6 to 9 and the minimum Hamming distance from 2 to 5 (Supplementary Figure 1), consequently decreasing the risk of crosstalk due to sequencing errors in the barcode (Wright and Vetsigian, 2016).

The first UDB approach implemented and evaluated, hereafter referred to as UDB-H11, is based on both the forward and reverse primers being modified to include the H1 sequence (analogous to the SB setup), and barcoding being performed with two distinct, unique bc12-H1 fusion primers (Figure 1). This barcoding approach reduces the crosstalk risk associated with single barcoding (Kircher et al., 2012; Esling et al., 2015), but only allows for the multiplexing of 274 samples amplified with the same primer pair into a single amplicon pool library (274 × *n* × *m* samples, if *n* distinct target-gene primer pairs are used and *m* amplicon pool libraries were combined on a sequencing run).

To increase the number of samples that can be amplified with the same primer pair and be multiplexed into a single amplicon pool library, while adhering to the unique dual barcoding principle with the stringent set of bc12 selected, we introduced a second 16 nt head sequence variant (H2). In this UBD setup, referred to as UDB-H12, the forward and reverse primers are modified to contain the H1 and H2 sequence, respectively. In the barcoding (second step) PCR, a unique bc12-H1 and a second, unique bc12-H2 fusion primer are used for sample barcoding (Figure 1). With the UDB-H12 setup, 548 samples amplified with a single primer pair can be multiplexed in one amplicon pool library (548 × *n* × *m* samples, if *n* distinct target-gene primer pairs are used and *m* amplicon pool libraries were combined on a sequencing run).

Finally, we implemented and evaluated a CDB setup, for which the forward and reverse primers are modified to contain the H1 and H2 sequence - like in UDB-H12, and a unique combination of bc12-H1 and bc12-H2 fusion primers is used for barcoding. However, the same bc12-H1 fusion primer is used for barcoding multiple samples, always in combination with a distinct bc12-H2 fusion primer, resulting in not unique, but combinatorially-unique dual-barcoded amplicons (CDB-H12; Figure 1). This approach allows for multiplexing up to 299,756 samples amplified with the same primer pair into a single amplicon pool library (299,756 × *n* × *m* samples, if *n* distinct target-gene primer pairs are used and *m* amplicon pool libraries were combined on a sequencing run). For this evaluation, we used a subset of barcode combinations available for the CDB approach to multiplex a maximum of 480 samples in a single amplicon pool library.

### The Effect of Barcoding Approach on Assignable Sequence Yield per Amplicon Pool Library

**FIGURE 2:**
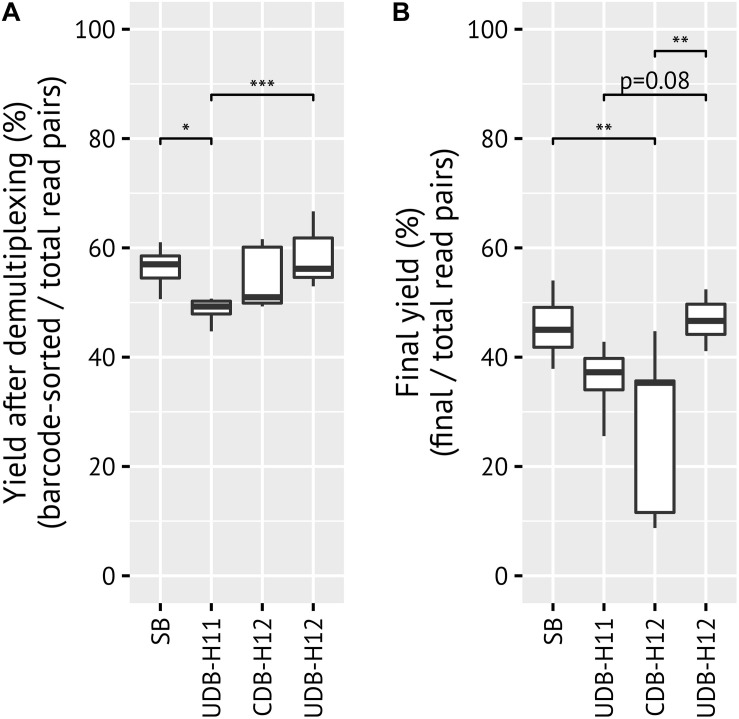
Comparisons of yield between barcoding systems. Yield describes the fraction of reads which matched to the expected barcode, linker, and primer by the total number of raw reads in each pool **(A)** before any quality filtering or denoising/clustering, and **(B)** after ASV inference and chimera filtering with DADA2. *p*-values > 0.1 not shown.

When comparing the yield per amplicon pool library, defined here as the fraction of reads that could be assigned to a sample *via* barcode matching, both CDB-H12 and UDB-H12 barcoding protocols performed comparably to previous results obtained with the SB approach (mean 54, 58, 56%, respectively), while UDB-H11 performed slightly worse (49%) (Figure 2A). The yield after ASV inference, i.e., after removal of low quality sequence reads, denoising and removal of chimeric sequences from each amplicon pool library, was comparable for the SB (46%, 38–54%), UDB-H11 (36%, 26–43%) and UDB-H12 (47%, 41–52%) setup, but significantly lower (Tukey multiple comparisons of means: *p* = 0.00441, 0.236, and 0.00178, respectively; −18.3, −9.1, and −19.6% difference in means, respectively; ANOVA: degrees of freedom = 3) and highly variable for the CDB-H12 setup (mean 27%, range 9–45%) (Figure 2B).

### Unique Dual Barcoding Minimizes Sample Crosstalk in Amplicon Pool Libraries

**FIGURE 3:**
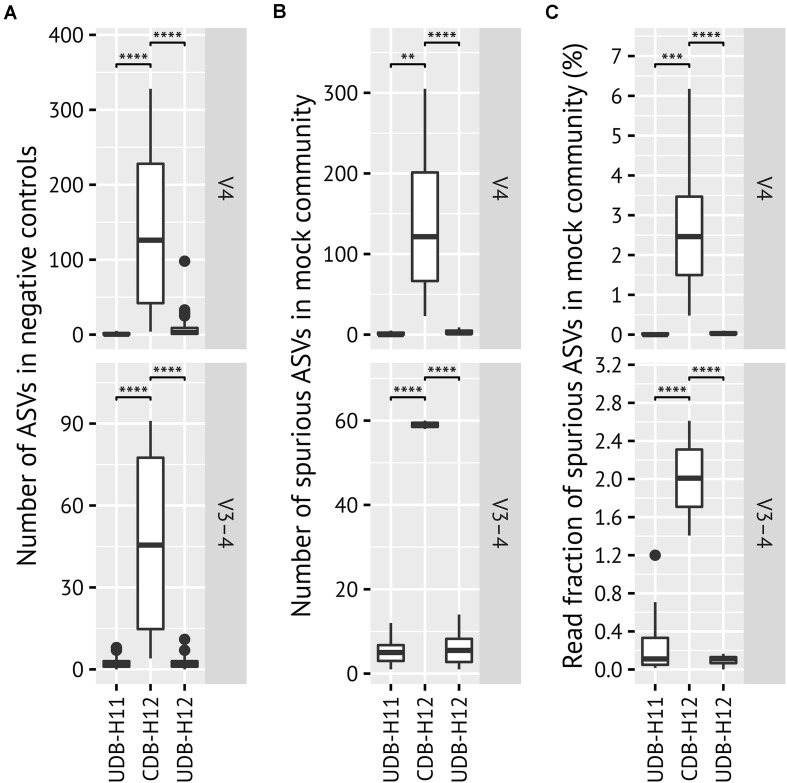
Evaluation of between-library contamination (crosstalk) based on **(A)** the number of ASVs detected in systematic PCR negative controls; **(B)** the number and **(C)** the fraction of spurious ASVs detected in mock community (ZymoBIOMICS Microbial Community DNA Standard II, D6311) amplicon libraries generated in two-step PCR procedure applying UDB-H11, CDB-H12 and UDB-H12 setups. Mock community members were identified using BLAST allowing for one mismatch. All other ASVs were defined as spurious (contaminants). All ASVs detected in systematic PCR negative controls are defined as spurious (contaminants). *p*-values > 0.1 not shown. *P*-value significance codes are *< 0.05, **< 0.01, ***< 0.001, and ****< 0.0001.

**FIGURE 4:**
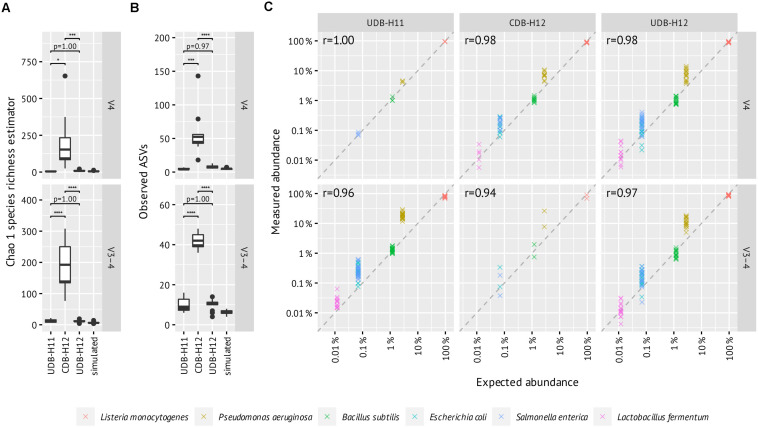
Evaluation of between-library contamination based on the alpha diversity of mock community (ZymoBIOMICS Microbial Community DNA Standard II, D6311) amplicon libraries generated in two-step PCR procedure applying UDB-H11, CDB-H12, and UDB-H12 setups and simulated count data based on the theoretical composition of 16S rRNA genes. **(A)** Chao 1 species richness estimator, **(B)** Observed ASVS, **(C)** Expected vs measured abundance of detected mock community members. All ASVs with one or zero mismatches to any of the reference sequences 16S rRNA genes are included and added up into the same species. Pearson’s *r* was calculated with log-transformed relative abundances per amplicon pool and then averaged. Enterococcus faecalis (expected 0.00067%) and Staphylococcus aureus (0.0001%) ASVs were sporadically detected between 0.006–0.05% abundance and were excluded due to their high likelihood of being cross-contaminations from gut and skin samples, respectively.

A noteworthy level of crosstalk between samples, defined as (i) the number of ASVs detectable in PCR negative control reactions, (ii) the number and fraction of spurious ASVs detectable in a commercially available mock community standard (ZymoMock), and (iii) the fraction of amplicon samples in which ASVs known to be specific to the mock community sample, was observed in the CDB-H12 setup, while close to no crosstalk was detectable in both UDB setups (Figure 3 and Supplementary Figure 2). Consequently, the species richness, expressed as Chao 1 species richness estimator, of amplicon libraries generated from the mock community standard (ZymoMock) barcoded with either of the two UDB setups (UD-H11 and UD-H12) matched well with the simulated species richness of the mock community standard (Figure 4A), while the species richness metric was inflated through the presence of spurious contaminants in samples barcoded with the CDB-H12 setup (Figure 4B). No significant difference in performance between the UDB-H11 and UDB-H12 setup was detectable.

To evaluate how well amplicon pool libraries produced with the different barcoding approaches reflect the relative abundances of the starting DNA pool, we correlated expected and measured relative abundances of the six most abundant mock community members (Figure 4C). This revealed similar patterns across all barcoding setups and primers, with little difference in correlation coefficient. Notably, *P. aeruginosa* was consistently overestimated in abundance, which may be due to primer bias (Schloss et al., 2011).

As no significant difference in data quality with regards to crosstalk between samples was observed between the UDB-H11 and UDB-H12 setup and UDB-H12 allows for a higher level of sample multiplexing, only the UDB-H12 and CDB-H12 approaches were further evaluated.

### Unique Dual Barcoding Minimizes Inter-Run Batch Effects

Finally, we evaluated the occurrence of batch effects in the UDB-H12 and CDB-H12 two-step PCR workflows. To this end, we compared the observed microbial community composition retrieved from replicate samples of a repeatedly sequenced (*n* = 22) complex environmental sample, barcoded with the CDB-H12 or the UDB-H12 setups and sequenced (i) within the same amplicon sequencing run, or (ii) in separate amplicon sequencing runs. Moreover, we evaluated the effect of sequence data processing on the occurrence and significance of batch effects. To this end, DADA2 error modeling and ASV inference was performed (i) using sequence data from all amplicon libraries generated on a sequencing run (i.e., the environmental control sample, mock community standard, and real sample data from other analysis projects), and (ii) using only sequencing data from the environmental control sample amplicon libraries. Finally, we evaluated the effect of spurious ASV filtering on between sample dissimilarity by removing ASVs that never exceeded the relative abundance threshold of 0.25% in the dataset (Reitmeier et al., 2020) prior to calculation of pairwise Bray-Curtis dissimilarity between samples.

**FIGURE 5:**
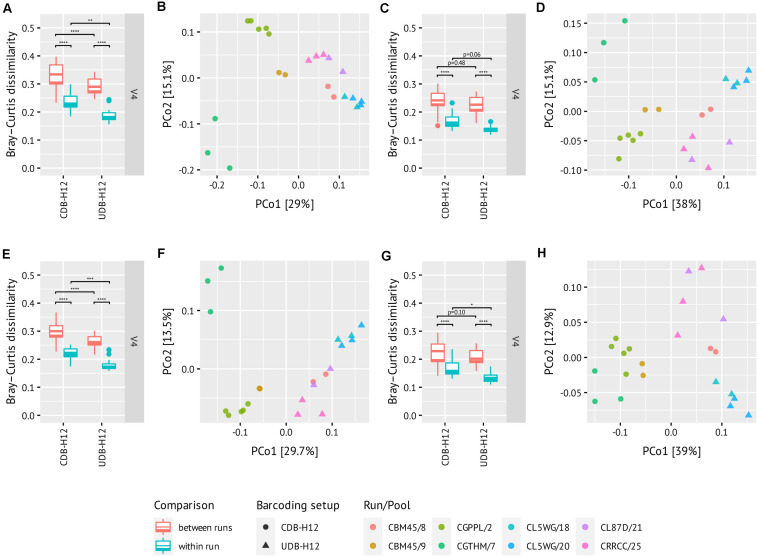
Pairwise distances boxplots **(A/C/E/G)** and oridations **(B/D/F/H)** between technical replicates of the same soil DNA. Libraries from the same run are of the same color. Amplicons were generated using the V4 primer set. Analyses were performed using all libraries of one run **(A–D)** or only the control samples **(E–H)** as input to DADA2 error modeling and pooled ASV inference. Abundance filtering of all ASVs never exceeding 0.25% was performed for **(C/D)** and **(G/H)**. Three runs per barcoding setup. Libraries were rarefied to the smallest read depth, i.e., **(A/B)**, 2792 reads; **(C/D)**, 2460 reads; **(E/F)**, 2861 reads; **(G/H)**, 2679 reads.

Regardless of the applied barcoding setup (UDB-H12 or CDB-H12) or data processing pipeline, the pairwise Bray-Curtis dissimilarity between samples barcoded with the same protocol and sequenced within the same run was always significantly lower than the dissimilarity between samples barcoded with the same protocol but sequenced on separate sequencing runs (Figure 5, Tukey multiple comparisons of means: *p* = 7.52e–14, 1.54e–13, 6.95e–12, 6.89e–13, 7.78e–14, 1.36e–13, 4.83e–08, and 5.64e–09, respectively; difference in means: −0.096, −0.10, −0.070, −0.088, −0.075, −0.081, −0.057, and −0.072, respectively; ANOVA: degrees of freedom = 1). Furthermore, the dissimilarity between samples barcoded with the UDB-H12 setup was significantly lower than the dissimilarity of samples barcoded with the CDB-H12 setup (Figure 5). While the amplicon library input mode for DADA2 error modeling and ASV inference (all amplicon libraries from one run vs. only amplicon libraries of the target sample) did not have an effect on the observed differences between barcoding setups (Figure 5A/E, Tukey multiple comparisons of means: *p* = 2.78e–05, 2.16e–03, 6.84e–06, 6.02e–04, respectively; difference in means: −0.040, −0.046, −0.034, and −0.040, respectively; ANOVA: degrees of freedom = 1), the removal of spurious ASV by abundance filtering, as expected, strongly reduced the difference in observed sample dissimilarity between the two barcoding setups (Figure 5C/G, Tukey multiple comparisons of means: *p* = 4.76e–01, 6.12e–02, 9.98e–02, and 2.82e–02, respectively; difference in means: −0.011, −0.029, −0.018, and −0.033, respectively; ANOVA: degrees of freedom = 1). These results indicate that abundance filtering of spurious ASVs is a suitable approach to significantly reduce effects of crosstalk between samples in combinatorial dual (and presumably single) barcoding approaches.

Notably, regardless of the barcoding protocol applied, batch effects associated either with amplicon PCR, sequencing library preparation, or sequencing were nonetheless still observed. While application of the UDB protocol as well as spurious ASV removal do reduce these effects, they do not completely eliminate them. Consequently, the occurrence and magnitude of batch effects must ultimately be taken into consideration and accounted for during amplicon pool library design, barcoding protocol selection, and data analysis.

## Conclusion

We established and benchmarked dual barcoding protocols based on a two-step PCR amplicon sequencing workflow (Herbold et al., 2015), and evaluated and compared their performance with regards to accuracy and reproducibility. Combinatorial dual barcoding with 12 nt barcodes allows for multiplexing orders of magnitude higher numbers of amplicon libraries of the same gene target, as long as low levels of crosstalk between samples are of no concern for the research goal. CDB is a promising approach for large-scale amplicon sequencing projects allowing for the cost-effective and time-efficient generation of datasets minimally affected by sequencing-associated batch effects, as hundreds of thousands of samples can be sequenced in a single run. However, for mid-scale amplicon sequencing projects, UDB barcoding strategies are the better choice, as lower levels of sample crosstalk and within-run and between-run dissimilarity between the inferred microbial community composition of replicate samples were observed. Overall, the here described workflow results in an improved consistency and reproducibility between amplicon sequencing runs, *via* an optimized sample barcoding approach, a standardized amplicon pool design and a new, superior amplicon sequence processing pipeline. The here presented dual barcoding two-step PCR workflows are currently implemented as standard operating procedures (SOPs) for amplicon sequence generation and analysis at the Joint Microbiome Facility (JMF) of the University of Vienna and the Medical University of Vienna^3^.

## Data Availability Statement

Datasets used in this study are deposited in the NCBI SRA (https://www.ncbi.nlm.nih.gov/sra/) under the BioSample accession numbers: SAMN16987472 (negative controls), SAMN13555796 (ZymoMock), and SAMN13555843 (environmental sample).

## Author Contributions

PP, BH, AL, and DB concieved the study. PP, JS, and GK performed experiments. PP, BH, and CH performed data analysis. PP and BH wrote the manuscript with support of all other authors. All authors contributed to the article and approved the submitted version.

## Conflict of Interest

The authors declare that the research was conducted in the absence of any commercial or financial relationships that could be construed as a potential conflict of interest.

## Footnotes

1 sourceforge.net/projects/bbmap

2 github.com/jfjlaros/demultiplex

3 https://jmf.univie.ac.at/

## Supplementary Material

Supplementary Figure 1 Hamming and Sequence Levenshtein distance between the newly introduced 548 12 nt barcodes (upper panel) and the 520 8 nt barcodes used for sample barcoding in the here evaluated two-step PCR approach. Distances were calculated using the R package DNABarcodes version 1.20.0 (Buschmann and Bystrykh, 2013).

Supplementary Figure 2 Detection of select mock community (ZymoBIOMICS Microbial Community DNA Standard II) ASVs in amplicon libraries generated from sample material. The selected organisms *Listeria monocytogenes*, *Pseudomonas aeruginosa*, and *Bacillus subtilis* account for 95.9, 2.8, and 1.2% of 16S rRNA gene copies in the ZymoBIOMICS Microbial Community DNA Standard II, respectively. *p*-values > 0.1 not shown.

Supplementary Table 1 Theoretical composition of the ZymoBIOMICS Microbial Community DNA Standard II (ZymoMock) as provided by the manufacturer (Genomic DNA, Genome copies, 16S rRNA gene copy number, 16S rRNA gene copies from Table 1 in the ZymoBIOMICS manual). In addition, simulated counts for V4 and V3–4 amplicons to a total read depth of 2,438 are given (as used in Figure 4).

Supplementary Table 2 Barcode sequences of applied 8 nt barcodes (5′–3′), sourced from a list of previously published 8 nt barcodes (Faircloth and Glenn, 2012), that were previously evaluated in 11.

Supplementary Table 3 Barcode sequences of applied 12 nt barcodes (5′–3′), selected from a list of 959 12 nt barcodes used in the Earth Microbiome Project (Herbold et al., 2015), according to following criteria: Hamming distance ≥ 4 from one another as determined by the R-package DNABarcodes (Hausmann et al., 2016), and together having a uniform base distribution at each barcode base position.

Supplementary Table 4 SRA accession numbers and details of all analyzed NC (negative controls), ZymoMock, and Soil (environmental control sample) libraries. Some amplicon libraries were assigned barcodes but yield zero reads (NC), and therefore were also not submitted and have no accession numbers.

## References

[B1] Allaband C. McDonald D. Vázquez-Baeza Y. Minich J. J. Tripathi A. Brenner D. A. (2019). Microbiome 101: studying, analyzing, and interpreting gut microbiome data for clinicians. *Clin. Gastroenterol. Hepatol.* 17 218–230. 10.1016/j.cgh.2018.09.017 30240894 PMC6391518

[B2] Andersen K. S. Kirkegaard R. H. Karst S. M. Albertsen M. (2018). ampvis2: an R package to analyse and visualise 16S rRNA amplicon data. *BioRxiv* [Preprint] 10.1101/299537 BioRxiv: 299537

[B3] Apprill A. McNally S. Parsons R. Weber L. (2015). Minor revision to V4 region SSU rRNA 806R gene primer greatly increases detection of SAR11 bacterioplankton. *Aquat. Microb. Ecol.* 75, 129–137. 10.3354/ame01753

[B4] Buschmann T. Bystrykh L. V. (2013). Levenshtein error-correcting barcodes for multiplexed DNA sequencing. *BMC Bioinformatics* 14:272. 10.1186/1471-2105-14-272 24021088 PMC3853030

[B5] Callahan B. J. McMurdie P. J. Holmes S. P. (2017). Exact sequence variants should replace operational taxonomic units in marker-gene data analysis. *ISME J.* 11 2639–2643. 10.1038/ismej.2017.119 28731476 PMC5702726

[B6] Callahan B. J. McMurdie P. J. Rosen M. J. Han A. W. Johnson A. J. A. Holmes S. P. (2016a). DADA2: high-resolution sample inference from Illumina amplicon data. *Nat. Methods* 13 581–583. 10.1038/nmeth.3869 27214047 PMC4927377

[B7] Callahan B. J. Sankaran K. Fukuyama J. A. McMurdie P. J. Holmes S. P. (2016b). Bioconductor workflow for microbiome data analysis: from raw reads to community analyses. *F1000Research* 5:1492. 10.12688/f1000research.8986.2 27508062 PMC4955027

[B8] Esling P. Lejzerowicz F. Pawlowski J. (2015). Accurate multiplexing and filtering for high-throughput amplicon-sequencing. *Nucleic Acids Res.* 43 2513–2524. 10.1093/nar/gkv107 25690897 PMC4357712

[B9] Fadrosh D. W. Ma B. Gajer P. Sengamalay N. Ott S. Brotman R. M. (2014). An improved dual-indexing approach for multiplexed 16S rRNA gene sequencing on the Illumina MiSeq platform. *Microbiome* 2 1–7. 10.1186/2049-2618-2-6 24558975 PMC3940169

[B10] Faircloth B. C. Glenn T. C. (2012). Not all sequence tags are created equal: designing and validating sequence identification tags robust to indels. *PLoS One* 7:e42543. 10.1371/journal.pone.0042543 22900027 PMC3416851

[B11] Gilbert J. A. Meyer F. Antonopoulos D. Balaji P. Brown C. T. Brown C. T. (2010). Meeting report: the terabase metagenomics workshop and the vision of an Earth microbiome project. *Stand. Genomic Sci.* 3 243–248. 10.4056/sigs.1433550 21304727 PMC3035311

[B12] Hamady M. Knight R. (2009). Tools, techniques, and challenges microbial community profiling for human microbiome projects. *Genome Res.* 19 1141–1152. 10.1101/gr.085464.108 19383763 PMC3776646

[B13] Hamady M. Walker J. J. Harris J. K. Gold N. J. Knight R. (2008). Error-correcting barcoded primers for pyrosequencing hundreds of samples in multiplex. *Nat. Methods* 5 235–237. 10.1038/nmeth.1184 18264105 PMC3439997

[B14] Hausmann B. Knorr K. H. Schreck K. Tringe S. G. Del Rio T. G. Loy A. (2016). Consortia of low-abundance bacteria drive sulfate reduction-dependent degradation of fermentation products in peat soil microcosms. *ISME J.* 10 2365–2375. 10.1038/ismej.2016.42 27015005 PMC4930147

[B15] Herbold C. W. Pelikan C. Kuzyk O. Hausmann B. Angel R. Berry D. (2015). A flexible and economical barcoding approach for highly multiplexed amplicon sequencing of diverse target genes. *Front. Microbiol.* 6:731. 10.3389/fmicb.2015.00731 26236305 PMC4503924

[B16] Herlemann D. P. Labrenz M. Jürgens K. Bertilsson S. Waniek J. J. Andersson A. F. (2011). Transitions in bacterial communities along the 2000 km salinity gradient of the Baltic Sea. *ISME J.* 5 1571–1579. 10.1038/ismej.2011.41 21472016 PMC3176514

[B17] Kircher M. Sawyer S. Meyer M. (2012). Double indexing overcomes inaccuracies in multiplex sequencing on the Illumina platform. *Nucleic Acids Res.* 40:e3. 10.1093/nar/gkr771 22021376 PMC3245947

[B18] Knight R. Vrbanac A. Taylor B. C. Aksenov A. Callewaert C. Debelius J. (2018). Best practices for analysing microbiomes. *Nat. Rev. Microbiol.* 16 410–422. 10.1038/s41579-018-0029-9 29795328

[B19] MacConaill L. E. Burns R. T. Nag A. Coleman H. A. Slevin M. K. Giorda K. (2018). Unique, dual-indexed sequencing adapters with UMIs effectively eliminate index cross-talk and significantly improve sensitivity of massively parallel sequencing. *BMC Genomics* 19:30. 10.1186/s12864-017-4428-5 29310587 PMC5759201

[B20] Oksanen J. Blanchet F. G. Friendly M. Kindt R. Legendre P. McGlinn D. (2020). *vegan: Community Ecology Package. R package version 2.5-7.* Available online at: https://CRAN.R-project.org/package=vegan (accessed February 19, 2021)

[B21] Parada A. E. Needham D. M. Fuhrman J. A. (2016). Every base matters: assessing small subunit rRNA primers for marine microbiomes with mock communities, time series and global field samples. *Environ. Microbiol.* 18 1403–1414. 10.1111/1462-2920.13023 26271760

[B22] Pollock J. Glendinning L. Wisedchanwet T. Watson M. (2018). The madness of microbiome: attempting to find consensus “best practice” for 16S microbiome studies. *Appl. Environ. Microbiol.* 84:e02627–17. 10.1128/AEM.02627-17 29427429 PMC5861821

[B23] Quail M. A. Smith M. Jackson D. Leonard S. Skelly T. Swerdlow H. P. (2014). SASI-Seq: sample assurance Spike-Ins, and highly differentiating 384 barcoding for Illumina sequencing. *BMC Genomics* 15:110. 10.1186/1471-2164-15-110 24507442 PMC4008303

[B24] R Core Team (2021). *R: A Language and Environment for Statistical Computing.* Vienna: R Foundation for Statistical Computing.

[B25] Reitmeier S. Hitch T. C. Fikas N. Hausmann B. Ramer-Tait A. E. Neuhaus K. (2020). Handling of spurious sequences affects the outcome of high-throughput 16S rRNA gene amplicon profiling. *Res. Sq. (preprint).* 10.21203/rs.2.21240/v1 PMC9723555 37938227

[B26] Schloss P. D. Gevers D. Westcott S. L. (2011). Reducing the effects of PCR amplification and sequencing artifacts on 16S rRNA-based studies. *PLoS One* 6:e27310. 10.1371/journal.pone.0027310 22194782 PMC3237409

[B27] Sinha R. Abu-Ali G. Vogtmann E. Fodor A. A. Ren B. Amir A. (2017). Assessment of variation in microbial community amplicon sequencing by the microbiome quality control (MBQC) project consortium. *Nat. Biotechnol.* 35 1077–1086. 10.1038/nbt.3981 28967885 PMC5839636

[B28] Walters W. Hyde E. R. Berg-Lyons D. Ackermann G. Humphrey G. Parada A. (2016). Improved bacterial 16S rRNA gene (V4 and V4-5) and fungal internal transcribed spacer marker gene primers for microbial community surveys. *Msystems* 1:e00009–15. 10.1128/mSystems.00009-15 27822518 PMC5069754

[B29] Wang Y. LêCao K. A. (2020). Managing batch effects in microbiome data. *Brief. Bioinform.* 21 1954–1970. 10.1093/bib/bbz105 31776547

[B30] Wright E. S. Vetsigian K. H. (2016). Quality filtering of Illumina index reads mitigates sample cross-talk. *BMC Genomics* 17:876. 10.1186/s12864-016-3217-x 27814679 PMC5097354

