# Supplementary figures and images for: An Economical and Flexible Dual Barcoding, Two-Step PCR Approach for Highly Multiplexed Amplicon Sequencing
Source: Front Microbiol. 2021 May 20;12:669776. doi: 10.3389/fmicb.2021.669776 (PMC8173057; doi:10.3389/fmicb.2021.669776)

Pairwise comparisons

Hamming

Sequence Levenshtein

8 nt barcodes

12 nt barcodes

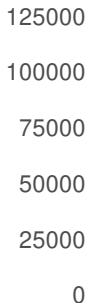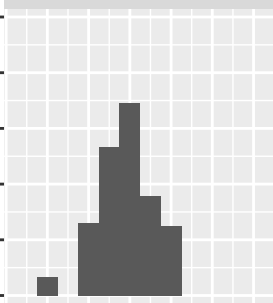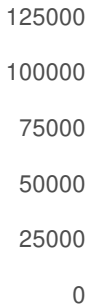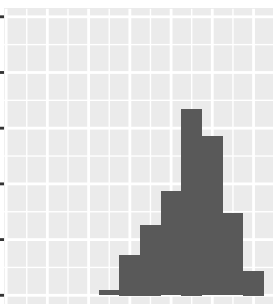

Distance

Supplement: Supplementary Figure 1 — Hamming and Sequence Levenshtein distance between the newly introduced 548 12 nt barcodes (upper panel) and the 520 8 nt barcodes used for sample barcoding in the here evaluated two-step PCR approach. Distances were calculated using the R package DNABarcodes version 1.20.0 (Buschmann and Bystrykh, 2013). [file Data_Sheet_1.PDF]

Libraries per pool in with ASVs  
of the mock community were detected (%)

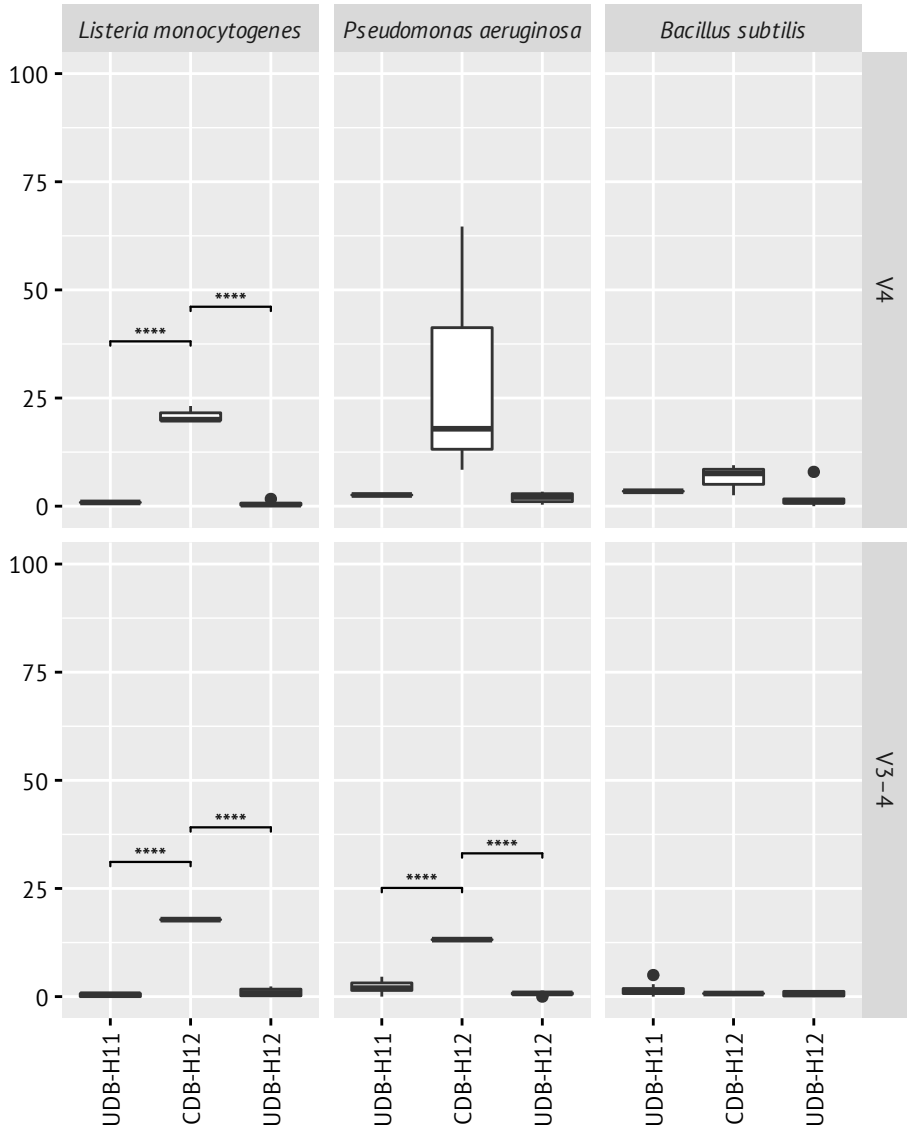

Supplement: Supplementary Figure 2 — Detection of select mock community (ZymoBIOMICS Microbial Community DNA Standard II) ASVs in amplicon libraries generated from sample material. The selected organisms Listeria monocytogenes, Pseudomonas aeruginosa, and Bacillus subtilis account for 95.9, 2.8, and 1.2% of 16S rRNA gene copies in the ZymoBIOMICS Microbial Community DNA Standard II, respectively. p-values > 0.1 not shown. [file Data_Sheet_2.PDF]
